# Royal Berkshire Hospital, Reading

**Published:** 1892-04-30

**Authors:** 


### The Institutional Workshop.

### WITHIN THE HOSPITALS.

### I.—ROYAL BERKSHIRE HOSPITAL, READING.

It has often been remarked that the hospital porter is a far more important personage to the well-being of a hospital than is generally recognised. Several correspondents have addressed us on this point in recent years, and we are not prepared to dispute the justice of the premises on which the opinion is based. Certainly the reception extended to visitors by the gate and hall porters at the Royal Berkshire Hospital has a tendency to influence him in favour of the institution. There is an aspect of quietude and efficiency about the approaches to this institution, which leads one to conclude that those responsible for its administration have the best interests of everyone at heart. Of course, a hospital may be so planned as to strike the visitor favourably or unfavourably. The site on which the Royal Berkshire Hospital stands is unusually good, and the whole aspect of the building, as viewed from the road, is attractive. Still, apart from this altogether, no stranger who visits this institution can fail to be favourably impressed by the general appearance of the interior arrangements, which will convince the experienced that the management is good. We were told that kindness rather than discipline formed the mainspring of the system on which the hospital is worked, everyone thus gradually coming to realise that they have a personal interest in the place, and so a feeling of attachment arises which is attended with the best results.

#### A Retrospective Glance.

This hospital was first opened in 1839, and although it has been considerably added to, especially of late years, it still presents many of the old features. It is interesting to walk over the buildings, to see the purposes to which the basements especially are now devoted, and to recall the uses to which they were put originally. Such a retrospective. glance is often the most effectual means of realising the progress that has been made in hospital administration Formerly laundry and washhouse were always in the basement, which also contained other matters that could only have a harmful effect upon the healthiness of the hospital wards. In the same way the offices attached to the various wards were badly situated and ill-adapted to their purpose. It was thought in these early days, and is still erroneously held by some, that each nurse should occupy a room immediately adjacent to her ward, from which she should be able to see what was going on within it. Of course such a contention conveys a very crude idea of the responsibilities attaching to a nurse's calling, and pays no regard whatever to the necessity for securing her against all avoidable health risks in her arduous calling. Painted metal baths, than which we know nothing less inviting or less adapted to hospital purposes, were for years in earlier days the only ones available; and we regret to see that the mistake of continuing them in the new nurses' quarters has been repeated in the recent extensions. Porcelain baths are the only suitable ones for use in a hospital, and no others should, under any circumstances, in our opinion, be permitted to be fixed for the use of patients or staff.

#### Suggested Improvements.

At the Royal Berkshire Hospital a new laundry and a nurses' home have been erected, and the ward offices have been placed for the most part outside and distinct from the wards. The laundry and nurses' home buildings are well adapted for the purposes to which they are put, but we should like to hear that the Committee had determined to replace the whole of the baths and slop sinks by more modern and suitable appliances than those now in use. Such a change would be calculated to improve the hygienic condition of the wards, and the cost would not be material to an institution which, we are glad to see, has a considerable amount of invested funds. In would further be a great improvement if arrangements could be made to transfer the kitchen to the top of the building on a plan similar to that adopted at the Liverpool Royal Infirmary, and also in the new wing at the Brompton Hospital for Consumption.

One great improvement of recent times has been the erection of a block of isolation wards in the grounds, apart from the hospital proper. These, we understand, have proved most useful, and have tended to increase the healthiness of the hospital.

#### In the Wards.

Sound general and economical considerations are evidently kept well in mind by the Managers of the Royal Berkshire Hospital. The wards are made as comfortable as circumstances will permit, and an intelligent regard for the patients' best interests has been shown by the erection, at the end of the principal wards, of large balconies into which the inmates can be wheeled. The balcony attached to the children's ward is quite a feature of the institution and must be of the greatest value and importance in securing the best results from medical treatment. We gathered that the head nurses have a free hand and are permitted to make their wards as comfortable and attractive as possible. For instance, in one of the men's wards there was quite a collection of mechanical toys, and it also contained birds, a large musical box, and an abundance of flowers. The birds were singing as we entered, and it may have been fancy, though we think not, but the aspect of the patients seemed to be brighter and more cheerful in this ward than elsewhere, although comfort seems to prevail throughout the hospital.

We were surprised at the number of patients in the Institution. The beds of most of the wards were full, and the children's ward was crowded as it contained at least two more beds than the cubic space available rendered desirable. This overcrowding could easily be avoided by the adoption of a rule which is usual in many general hospitals, viz., the exclusion of infants, *i. e.*, children under two years of age. It is not fair to the staff nor to the interest of the infants and their parents to admit such young children into a ward of a general hospital where they often interfere with the comfort and progress of other patients. No doubt this point has only to be mentioned in order to secure the adoption of some such rule as we have indicated. Some of the beds struck us as a little out of date, and we should strongly recommend the authorities to gradually introduce spring mattresses throughout the establishment.

The floors of the wards are polished, and the general aspect not only of the floors but of the furniture, brass-work, and various appliances, afforded evidence of the efficiency of the administration, which is in the charge of the secretary and house steward, Mr. John T. Hugo, and the Matron and Superintendent of nursing, Miss Baster.

#### How to Deal with Critics.

It was refreshing to notice the common sense and the kindly feeling which seems to prevail. It has long been the custom to deal with honest critics in a practical fashion, which we commend to the consideration of other committees. If a gentleman displays an interest in the hospital and becomes critical in his remarks, providing he is eligible, an early opportunity is found to invite him to become a member of the board. In this way useful members are often found, and the whole efficiency of the institution is increased.

#### Pensions.

Soon after the Royal National Pension Fund for Nurses was established, the Committee of this hospital considered the matter, and determined to commence a system inculcating thrift amongst the nurses. The one at present in force is not popular for obvious reasons, but it is a step in the right direction, and we have little doubt that in due course arrangements will be made whereby the nurses will enjoy the whole of the benefits which affiliation with the Royal National Pension Fund for Nurses would secure for them.

### Provision for Accidents.

There is an ambulance carriage used solely for the conveyance of patients to and from the hospital, which is sent out in cases of accident or emergency in any direction not exceeding a radius of fifteen miles from Reading. When necessary a nurse accompanies it, and, where they are in a condition to do so, the friends are called upon to defray the cost for horse hire. The House Surgeon, Mr. E. Ward, decides in each case whether the ambulance shall be sent or not.

### The Convalescent Fund.

There is a Convalescent Fund, administered by the Board of Management, which we commend to the attention of other hospital committees. This fund is applied to the relief of patients on the recommendation of the medical staff. It provides surgical instruments for such patients as have not the means of procuring them ; and gives pecuniary help to the poorest patients, either directly to defray the cost of their transit home, or to provide temporary relief to render permanent any benefit they may have received from hospital treatment. This fund also secures convalescent aid for the patients, and defrays the cost. Last year, of the total sum expended, 6½ per cent. was given in relief to patients, 22 per cent. was devoted to the purchase of instruments, and 71½ per cent. defrayed the cost of sending patients to and treating them in convalescent institutions.

### Finances.

Steadily and successfully the Managers have gradually husbanded the resources of the institution until they have accumulated invested property to the extent of upwards of £40,000 ; and we are glad to note that the inhabitants of the district which this hospital serves show an intelligent appreciation of the good management of the Royal Berkshire Hospital by the display of steady liberality in donations and subscriptions. The ordinary income amounted to £8,486 in 1891, or £2,100 more than in 1890, and this has enabled the Committee to meet the necessary expenditure and to pay off a debt of £438 for the previous year. All these facts are satisfactory, and we congratulate the Board upon the present condition of prosperity and efficiency to which the hospital has attained.

### Nursing.

Miss Baster, whose twenty years' faithful service is, we hope, appreciated, has become quite a mine of information, and anyone interested in hospitals, especially some of our modern enthusiasts, would do well to make her acquaintance and so secure a pleasant and profitable experience. There was a joy in witnessing the pleasure which is given by the new nurses' dining-room, excellent as it is in all respects, and testifying to the cleverness of the architect and the enthusiasm of all who took part in the work, which did one more good than a week at the seaside. Real genuine enthusiasm in good work done for others is always refreshing, and Miss Baster and the staff of the Royal Berkshire Hospital possess this gift to an extent which must make its weight felt for good throughout the whole establishment. Many people like to encourage those who are doing really good work, and there is an opportunity at the present time for one or more friends to come forward and present the nurses with a piano for their sitting-room. This we hope will be forthcoming before Whitsuntide, for surely amongst the readers of The Hospital there must be more than one or two people who would gladly give a piano to such an institution as we have been describing for the relaxation of the nursing staff. We would, however, venture to remind them that they must act promptly or they will be sure to be supplanted by one or more friends of the hospital who appreciate its work and have only to hear of its requirement to promptly supply it.

### An Excellent Site.

Wealthy philanthropists who desire to give a site for a new hospital, and architects too, should go to Reading and walk over the grounds of the Royal Berkshire Hospital. It is admirably situated ; there is an abundance of garden and lawn space, the latter being utilised in the summer months with great advantage to the patients, whose beds are brought out whenever the weather permits. These grounds contain a special garden for the Matron, but we gathered that it was not much used, as it is a little out of the way. Still, it was a kindly thought to provide such a garden, and we commend the fact to other country hospitals. Ample space, freedom from other buildings, abundance of air and light, a pleasant prospect, and room for future extensions, are all essential points to remember in selecting a site for a hospital. Of course the site at Reading was selected before the town had attained to anything like the proportions it has at present, but it is satisfactory to note that the well-to-do people, if we may judge from the houses which surround the hospital, regard it as a pleasant neighbour, and one in no sense to be avoided. This latter fact is in itself equivalent to a revolution in public feeling in regard to hospitals, and proves that that feeling at Reading is not confined to the patients, but that it has permeated all classes of society.

### The Library.

In the days when the Royal Berkshire was first erected the medical profession were mainly dependent upon the hospital for their information in regard to the progress of medical science. Hence it was customary to erect a library building in connection with hospitals of this earlier period, which, in addition to the library proper, usually contained accommodation for the holding of meetings of medical societies, and a museum. Several of the libraries connected with county infirmaries are very valuable, although in many institutions they have been allowed to fall behind the times. The library at Reading is, however, well preserved, and although some of the more modern treatises may be missing, there has evidently been an attempt to keep it well up to date, and to secure that it shall be capable of really meeting the requirements of the medical profession of the neighbourhood. We hope to devote some space on another occasion to this and other libraries, but any account of the Royal Berkshire Hospital would be incomplete which omitted a word of praise to the excellent library, and to those who are responsible for its management.

